# Polymorphisms and mutations of ACE2 and TMPRSS2 genes are associated with COVID-19: a systematic review

**DOI:** 10.1186/s40001-022-00647-6

**Published:** 2022-02-22

**Authors:** Jingwei Li, Yali Wang, Yong Liu, Ziqu Zhang, Yuyun Zhai, Yan Dai, Zijian Wu, Xiang Nie, Lunfei Du

**Affiliations:** 1 Chengdu University of Traditional Chinese Medicine, Chengdu, Sichuan China; 2 The Third People’s Hospital of Zigong, Zigong, Sichuan China; 3 Zigong Hospital of Traditional Chinese Medicine, Zigong, Sichuan China; 4 Present Address: The Third People’s Hospital of Zigong, Zigong, Sichuan China

**Keywords:** ACE2, COVID-19, Genetic susceptibility, SARS-CoV-2, TMPRSS2

## Abstract

**Objective:**

To determine the effect of polymorphisms and mutations in angiotensin-converting enzyme 2 (ACE2) and Type 2 transmembrane serine proteases (TMPRSS2) genes on susceptibility to corona virus disease 2019 (COVID-19) and patient prognosis.

**Introduction:**

From December 2019 to the current time, an outbreak of epidemic of COVID-19, characterized by severe acute respiratory syndrome coronavirus 2 (SARS-CoV-2) has occurred around the world. It is now clear that SARS-CoV-2 binds to human ACE2 receptors, with expression of these receptors correlated with the rate of SARS-CoV-2 infection and mortality. Polymorphisms in individual patient factors, such as ACE2 and TMPRSS2 genes have been linked with an increase in negative outcomes, although evidence to affirm remains debatable.

**Methods:**

Here, we performed a systematic review, based on guidelines of the Preferred Reporting Items for Systematic Reviews and Meta-Analyses (PRISMA) criteria, with the aim of assessing whether polymorphisms in ACE2 and TMPRSS2 genes affect the COVID-19 condition. We extensively searched PubMed, MEDLINE, Embase, the Cochrane Library, and Web of Science databases, for relevant articles and reports published in English between December 2019 and December 2021.

**Results:**

A total of 495 full-text articles were downloaded, of which 185 were excluded after preliminary examination as they were duplicates. Finally, 310 articles were evaluated, by reading their titles and abstracts, and 208 of them eliminated based on our selection criteria. Finally, 33 articles met our inclusion criteria and were included in the final assessment. Genetic data from 33,923 patients with COVID-19 drawn from the general population and deriving from over 160 regions and 50 countries, as well as approximately 560,000 samples from global-public genetic databases, were included in our analysis. Ultimately, we identified 10 SNPs and 21 mutations in the ACE2 gene, along with 13 SNPs and 12 variants in the TMPRSS2 gene, which may be associated with COVID-19.

**Conclusions:**

ACE2 and TMPRSS2 play vital roles in the onset, development, and prognosis of SARS-CoV-2 infection, and have both been strongly associated with vulnerability, intensity, and the clinical result of COVID-19. Overall, these genetic factors may have potential for future development of personalized drugs and vaccines against COVID-19.

*Trial registration:* CRD42021239400 in PROSPERO 2021.

## Introduction

Severe acute respiratory syndrome coronavirus 2 (SARS-CoV-2), which was first discovered in December 2019 as a new human pathogen, has subsequently become a global pandemic [1]. As of December 25, 2021, over 279 million people had been diagnosed with Corona Virus Disease 2019 (COVID-19), of which 5.4 million had died [2]. Individuals infected with SARS-CoV-2 exhibit a broad spectrum of clinical symptoms, including fever, cough, and dyspnea, while less common symptoms are constituted by fatigue, headache, anosmia, ageusia, cutaneous manifestation, and gastrointestinal symptoms, whereas, another proportion prove entirely asymptomatic [3–5]. Notably, the severity of COVID-19 varies among individuals, and could range from mild flu-like symptoms to pneumonia, acute respiratory distress syndrome (ARDS), and death. Furthermore, there appears to be sex and age-related differences [6, 7]. Previous studies have demonstrated that manifestations and the course of COVID-19 disease are associated with an individual's age, race, ethnic origin, sex, angiotensin-converting enzyme 2 (ACE2) expression pattern, and immunological modulation [8]. Whether angiotensin-converting enzyme inhibitors (ACEI) and angiotensin receptor blocker (ARB) analogs are harmful when used in patients with COVID-19 who have hypertension is not currently in question. Researchers supporting the use of these drugs argue that ACE2 functional blockers may inhibit cellular entry of SARS-CoV-2 virus and thus improve patient prognosis. On the other hand, the opposing camp holds that continued use of these agents leads to high expression of ACE2 receptors in the respiratory epithelium, facilitating entry of SARS-CoV-2 into cells, which leads to enhanced viral replication and accompanying tissue damage [9, 10]. Therefore, understanding the inter-individual variability in relation to the disease is imperative to management. Epidemiological and genome-wide association studies have associated genetic variability to individual variations in sensitivity to COVID-19 [11].

The COVID-19 disease might be influenced by angiotensin-converting enzyme (ACE), ACE2, and Type 2 transmembrane serine proteases (TMPRSS2) genotypes, with previous studies reporting that allele frequencies and single nucleotide polymorphisms (SNPs) are correlated with individual variations in prevalence of COVID-19 among ethnic groups [12]. Particularly, male patients were found to be more substantially impacted than their female counterparts, due to variations in the genetic alterations of ACE-2 receptors, as shown by age-adjusted COVID-19 hospitalization and death rates [13]. Recent studies have also indicated that people with blood groups A and O are more vulnerable to COVID-19 [14]. Furthermore, numerous investigations have demonstrated the histology and pathophysiology of the SARS-CoV-2 illness. On the other hand, the spike glycoprotein (S1) has been shown to interact with ACE-2 receptors on host cell epithelial cells. For example, results from a previous experiment, based on nano-luciferase, revealed that the virus's ACE-2 receptor-binding protein has an extremely high affinity for the receptor [15]. For the SARS-CoV-2 particle to infect human lung epithelial cells, it requires the metallocarboxypeptidase (TMCP) ACE-2, while inhibition of renin–angiotensin system (RAS) caused ACE-2 to protect internal organs by degrading its substrate angiotensin II [16]. The human enzyme, TMPRSS2, processes the viral spike protein, and exposes it to the host’s ACE-2 binding site, which recognizes the S2 subunit's fused amino acid [17]. The role of TMPRSS2 in SARS-CoV-2 infection is primarily due to S-protein processing and priming [18]. In addition, SARS-CoV-2 subsequently uses cysteine proteases, such cathepsin B and L (Cat B/L), to facilitate fusion of the viral membrane to those of the hosts [19]. Evidence from a pool of miRNA-based research has suggested that TMPRSS2 could be a potential regulator of SARS-CoV-2 entrance checkpoint [20].

In this study, we hypothesized that ACE2 and TMPRSS2 may be influencing progression of COVID-19 disease, while various genetic patterns may be affecting the risk of infection, viral invasion, aggressiveness, and mortality rates of COVID-19. Thus, we systematically reviewed published literature describing genetic features of vulnerability and prognosis for COVID-19, with focus on polymorphisms of ACE2 and TMPRSS2 genes. Overall, our findings are expected to provide an understanding into the role of these genetic factors in COVID-19 and guide future development of treatment and prognosis approaches.

## Methods

This study examined the most recent evidence of genetic susceptibility to COVID-19 conducted before June 2021. The systematic review was conducted in accordance with the Preferred Reporting Items for Systematic Reviews and Meta-Analyses (PRISMA) guidelines [21].

### Data sources

Systematic literature searches were performed in MEDLINE, Embase, the Cochrane Library, and Web of Science databases, from their inception until December 25, 2021. Details of the search strategy are outlined in Table 1. Additionally, references for relevant reviews and publications that met the selection criteria were checked, subject specialists consulted, and Open Grey searched for additional texts.

**Table 1:** Search strategies used in the systematic review, described by databases to be searched

Search terms	Database
(((ace2) OR (tmprss2)) AND (gene polymorphism)) AND (covid-19)	MEDLINE(PubMed)
(ace2:ti,ab,kw OR tmprss2:ti,ab,kw) AND 'gene polymorphism':ti,ab,kw AND 'covid 19':ti,ab,kw	Embase
(ace2):ti,ab,kw OR (tmprss2):ti,ab,kw AND (gene polymorphism):ti,ab,kw AND (covid-19):ti,ab,kw (Word variations have been searched)	Cochrane
(((TS = (ace2)) OR TS = (tmprss2)) AND TS = (gene polymorphism)) AND TS = (covid-19)	Web of Science

### Selection criteria

Two independent investigators reviewed and selected papers based on their titles and abstracts. Articles were only selected if they met our set criteria, after examining the entire text of the retrieved documents. Only peer-reviewed original articles that were written in English and met our criteria were examined in the final report. Selection criteria used in this investigation were as follows:

#### Inclusion criteria

- A cohort study conducted to identify polymorphisms in ACE2 or TMPRSS2 genes obtained from COVID-19-positive patients using polymerase chain reaction.
- Genomic data at a population-wide scale.
- Studies that described a regimen for the treatment of COVID-19 and applied pharmacogenomic models.
- Studies that sought to identify genetic predictors of COVID-19 exposure, aggravation, and prognosis through the identification of SNPs associated with disease intensity or vulnerability.

#### Exclusion criteria

- Studies in which ACE2 and TMPRSS2 genes were neither sequenced nor their polymorphisms analyzed.
- Studies in which outcome variables were not correlated with severity or susceptibility of COVID-19 patients.
- Review articles.

### Data extraction

Two independent investigators read through the article and retrieved information, including article type (e.g., clinical research), author information, country of origin, sample size, genetic susceptibility data, and significant findings, then transferred to an organized sheet. The other writers cross-checked texts of all the selected papers to verify that there were no overlaps or duplications.

### Quality assessment

We adopted the Cochrane risk of bias tool to evaluate quality of the included studies [22]. The reviewer who extracted the data also performed a quality assessment during the extraction process. Next, two independent researchers assessed quality and consistency of each article, as well as the potential of bias. Any disagreements between them were resolved through a discussion with a third researcher. Finally, the complete text of selected publications was reviewed, and key results extracted.

## Results

**Fig. 1:**
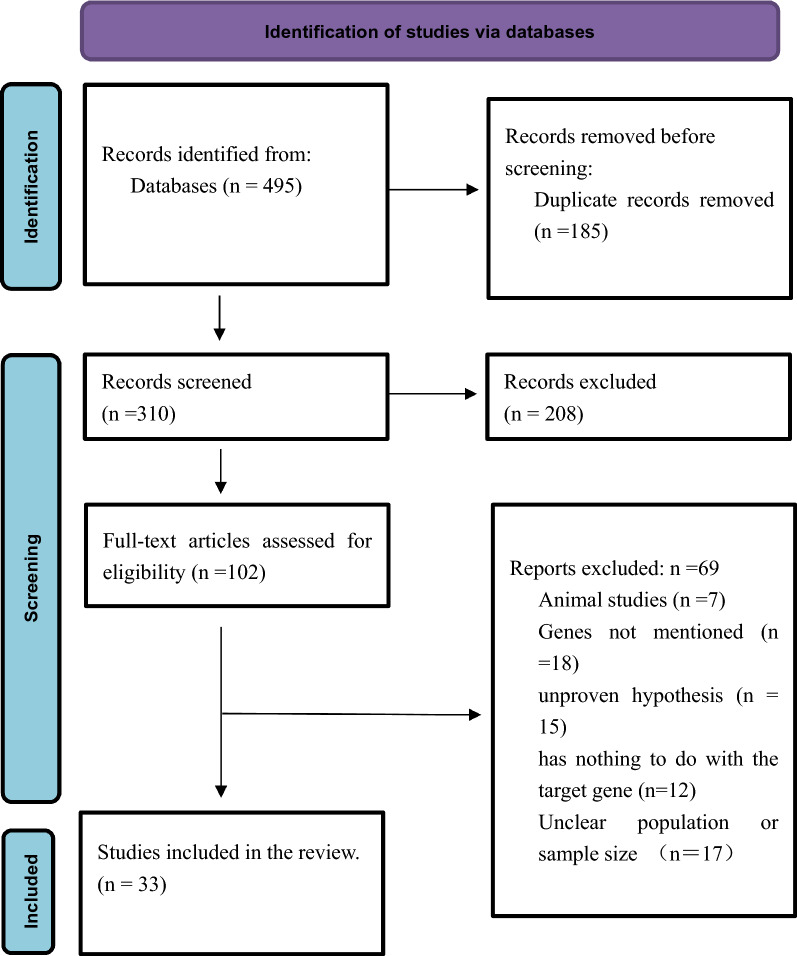
Flow diagram showing the selection process of identified articles

The aforementioned search strategy resulted in 495 articles, of which 185 were excluded since they were duplicates. Titles and abstracts of the remaining 310 papers were evaluated, and 208 excluded. The remaining 102 articles were taken through full-text evaluation, and ultimately 33 of them met our inclusion criteria and were therefore reviewed (Fig. 1). Results of the characteristics from the included studies are summarized in Table 2. Genetic data from 33,923 patients with COVID-19 drawn from the general population and deriving from over 160 regions and 50 countries, as well as approximately 560,000 samples from global-public genetic databases, were included in our analysis. Ultimately, we identified 10 SNPs and 21 mutations in the ACE2 gene, along with 13 SNPs and 12 variants in the TMPRSS2 gene, which may be associated with COVID-19. Overall, polymorphisms in ACE2 and TMPRSS2 genes influence susceptibility, symptoms, and severity of patients to COVID-19 among populations across different regions of the world, suggesting that these genes could be potential targets in development of treatment therapies for COVID-19.

**Table 2:** Characterization of the included studies

ID	Name of the first author	Country	Type of study	Study population	Gene loci and polymorphism	Sample size	Study design
1	Aleksei Zarubin [30]	Russia	Research study	COVID-19 patients and normal people from Eastern Europe and North-East Asia	TMPRSS2	1836	Descriptive research
2	Xia Xue [48]	China	Research study	SARS-CoV-2 genomic sequences from 160 regions and 50 countries	ACE2	2092	Ecological study
3	LaksmiWulandari[36]	England	Primary research	COVID-19 patients	TMPRSS2 p.Val160Met	95	Cohort study
4	Lars Wallentin[49]	Sweden	Clinical research	Two international cohorts of elderly patients with atrial fibrillation	ACE2	5087	Cohort study
5	Gilberto Vargas-Alarcón[34]	Mexico	Research study	The 1000 Genome Project comprising four populations from America, Africa, Europe, and Asia	ACE2, TMPRSS2, TMPRSS11A, ELANE, and CTSL	613	Cohort study
6	Laura Torre‐Fuentes[29]	Spain	Research study	A 120-person familial Multiple Sclerosis (MS) cohort from Madrid	ACE2, TMPRSS2, and Furin	138	Descriptive research
7	Mukesh Thakur[47]	India	Research study	COVID-19 patients	ACE2	112	Descriptive research
8	Kushal Suryamohan[26]	USA	Research study	290,000 samples from available genomic databases	ACE2	290,000	Descriptive research
9	Claudia Strafella[31]	Italy	Research study	Healthy individuals from Italy	ACE2	268	Cohort study
10	Anshika Srivastava[33]	India	Research study	393 samples obtained globally, with an emphasis on South Asia	ACE2	393	Descriptive research
11	Maria K. Smatti[50]	Qatar	Primary research	Participants in Qatar Biobank	TMPRSS2	6218	Original research
12	Anton E. Shikov[37]	Russia	Original research	COVID-19 patients and healthy individuals in Russia	ACE2	1359	Original research
13	Kristina Schönfelde [51]	Germany	Research study	COVID-19 patients and healthy individuals in Germany	TMPRSS2	492	Case–control study
14	Christopher P.Nelson[52]	UK	Research study	Cohorts of BIOSTAT -CHF	ACE2	3442	Relevance study
15	Maria Monticelli[39]	Italy	Research study	COVID-19 patients in Italy	TMPRSS2	1177	Relevance study
16	Birte Möhlendick[41]	Germany	Original research	COVID-19 patients and healthy individuals in Germany	ACE2	550	Relevance study
17	Esteban A. Lopera Maya[53]	Netherlands	Original research	Healthy people in northern Netherlands	ACE2 and TMPRSS2	36,339	Relevance study
18	Javier Martínez-Sanz[25]	Spain	Original research	39 inpatients and 28 uninfected but heavily exposed healthcare personnel	ACE2	67	Relevance study
19	Andrea Latini[27]	Italy	Research study	COVID-19 patients	TMPRSS2	131	Cohort study
20	Hossein Lanjanian[28]	Iran	Research study	Participants from Tehran Cardio-Metabolic Genetic (TCGS)	ACE2	3704	Cohort study
21	Andre' Salim Khayat[54]	Brazil	Research study	Amerindians and interbred people in northern Brazil's Amazon region	ACE2	2650	Cohort study
22	Yuan Hou[45]	USA	Research study	81,000 human genomes all over the world	ACE2 and TMPRSS2	Approximately 81,000	Relevance study
23	Pavel HAMET[46]	Canada	Clinical research	French-Canadian and British populations	ACE2	780	Cohort study
24	Xingyi Guo[55]	USA	Research study	People from the GAD (gnomAD v2.1.1)	ACE2	15,708	Relevance study
25	Juan Gómez[56]	Spain	Clinical research	COVID-19 patients and ordinary people in Spain	ACE, ACE2	740	Cohort study
26	Liam Gaziano[57]	USA	Research study	COVID-19 inpatients and ordinary people	ACE2, IFNAR2, IL-10Rb	7554	Cohort study
27	David Curtis[58]	UK	Research study	82 COVID-19 patients and normal 49,953 people in the UK Biobank	ACE2 and TMPRSS2	50,035	Cohort study
28	Concetta Cafiero [35]	Italy	Original research	COVID-19 patients (symptomatic and asymptomatic both included)	ACE, ACE2, AGT, and AGTR1	104	Cohort study
29	Rosanna Asselta[12]	Italy	Research study	COVID-19 patients and normal people in Italy	ACE2 and TMPRSS2	3984	Cohort study
30	Immacolata Andolfo[43]	Italy	Research study	COVID-19 hospitalized patients	MX1 and TMPRSS2	6406	Cohort study
31	Hui Liu[59]	China	Research study	Independent SNPs from the GSCAN consortium	ACE2	532	Descriptive research
32	Sungwon Jeon[44]	Korea	Research study	72,907 samples from 29 countries	TMPRSS2 V197M	72,907	Cohort study
33	Mohammad Muzaffar Mir[60]	Saudi Arabia	Clinical research	117 consecutive COVID-19 patients and 150 age matched healthy controls	ACE2	267	Case–control study

## Discussion

Previous studies have shown that cell–virus interaction involves connecting a transmembrane glycoprotein spike (S), which exists in the viral surface in the form of trimers, to ACE2 [23]. Influenza and coronaviruses significantly depend on the TMPRSS2 gene for viral entry and spread inside a host, using similar mechanisms. As soon as the virus's viral hemagglutinin protein reaches respiratory epithelial cells, it attaches to the ACE2. Histamine is then cleaved to induce viral internalization at a later phase. This second phase requires TMPRSS2, a type 2 transmembrane serine protease [24].

In the literature included, we isolated 10 SNPs and 21 mutations in the ACE2 gene and 13 SNPs and 12 mutations in the TMPRSS2 gene potentially related to COVID-19. COVID-19 may be affected by these polymorphisms or variants in terms of virus invasion, population distribution, disease severity, complications, mortality, and treatment.

First of all, polymorphisms and variations in the ACE2 and TMPRSS2 genes may impact SARS-COV-2 virus invasion and population susceptibility to the infection. In a Spain study involving 28 uninfected but highly exposed healthcare workers and 39 hospitalized COVID-19 patients, two TMPRSS2 variants proved to be linked to an increased likelihood of SARS-CoV-2 susceptibility: the minor A allele in the rs2106806 variant in addition to the minor A allele in the rs2106807 variant and the minor T allele in the rs6629110 variant [25]. Another US study discovered that the S-protein affinity of K31R and E37K variations proved to be lower than that of wild-type ACE2, whereas K26R and T92I variants proved higher. Soluble ACE2 K26R and T92I were demonstrated to be more effective in inhibiting S-protein pseudo-typed viral entry, indicating that ACE2 variants may potentially affect SARS-CoV-2 susceptibility [26]. The frequencies of the TMPRSS2 variant alleles c.331G > A, c.23G > T, and c.589G > A in an Italian cohort of COVID-19 patients were demonstrated to vary significantly from the corresponding allele frequencies in the GnomAD database. Genetic variation in these genes bears the potential of influencing SARS-CoV-2 entry [27] In addition, in a Tehran cardiometabolic genetic study, two ACE2 gene missense mutations, K26R and S331F, were found to reduce the receptor's affinity for the viral Spike protein. Moreover, a critical role for ACE2 Arg652 in TMPRSS2 protease function was also discovered, employing bioinformatic modeling of three-dimensional structure along with protein docking [28]. Despite the fact that TMPRSS2 proves widely polymorphic in the Madrid familial multiple-sclerosis cohort, only rs75603675 has been linked to SARS-CoV-2 infection. Furthermore, a link between the synonymous variants rs61735792 and rs61735794 and the disease was established [29].

In variant regions and populations, statistical significance is found to exist in certain mutations and polymorphisms. Additionally, a functionally significant missense mutation in exon 6/7 of the TMPRSS2 gene has been demonstrated to be highly common in numerous human groups, with a region-specific distribution pattern. Notably, the prevalence of missense mutations represented by rs12329760 varied between 10 and 65%, with Asian populations showing a significantly higher frequency than European populations [30]. In Italy, the general population shows a significant variance from that of the global population in terms of the frequency distribution of 2 SNVs (rs35803318 and rs2285666) [31]. Furthermore, in an ACE2 research, it was discovered that most South Asian haplotypes are more closely related to East Eurasians than to West Eurasians. According to phylogenetic study, the South Asian haplotypes shared by East Eurasians prove to be composed of two distinct event polymorphisms (rs4646120 and rs2285666). Thus, host sensitivity to the new coronavirus SARS-CoV-2 may in fact prove closer to that of East Asians than to that of Europeans in South Asians [32, 33]. One study examined the allele frequencies of the ACE2, TMPRSS2, TMPRSS11A, cathepsin L (CTSL), and elastase (ELANE) genes in four populations from the American, African, European, and Asian continents identified a potentially disruptive polymorphism in the TMPRSS2 gene (rs12329760), the minor allele frequencies of which varied between populations [34]. In African and Eastern Mediterranean people, polymorphisms in the ACE2 gene were reported to offer protection against development of COVID-19 [35].

Actually, the overwhelming majority of research we reviewed indicated that polymorphisms or mutations in both genes proved strongly linked with either positive or negative impacts on COVID-19 severity and mortality. Notably, a study of the Indian population identified a probable association between the TMPRSS2 pVal160Met polymorphism and SARS-CoV2 infection and COVID-19 results [36]. In a population of Russian patients, the influence of numerous types of ACE2 variations on COVID-19 outcomes indicated that common missense and regulatory variants could not account for illness-severity disparities. Some unusual ACE2 variations (such as rs146598386, rs73195521, and rs755766792) may influence COVID-19 outcomes [37]. In a German case–control investigation, the CC genotype of TMPRSS2 rs383510 demonstrated relations, with a 1.73-fold greater risk of SARS-CoV-2 infection but not, however, with COVID-19 severity. Neither the TMPRSS2 rs2070788 nor rs12329760 polymorphisms showed a demonstrable relation with an increased risk of SARS-CoV-2 infection or severity of COVID-19. Furthermore, the rs383510 CC genotype remained an independent predictor of a twofold higher probability of SARS-CoV-2 infection in multivariate analysis (MVA)[38]. In the Italian COVID-19 patient population, the predominant mutation p.V197M showed a detrimental effect on proteases but was found beneficial to patients (rs12329760). Variations prove to be more abundant in individuals bearing concurrent disease who do not require hospitalization or oxygen therapy than in individuals who do require oxygen therapy, ventilation, or intubation [39, 40]. According to a German study, the ACE2 rs2285666, GG genotype, or G allele proved to be related, with a nearly twofold greater risk of SARS-CoV-2 infection and a threefold increased risk of serious disease or death, due to COVID-19 [41]. In an Italian investigation, it was discovered that the SNPs rs2074192 (ACE2), rs1799752 (ACE1), and rs699 (AGT) proved serviceable for predicting clinical outcome in SARS-CoV-2 infected people [35]. Severe COVID-19 was shown to be related with five SNPs in the TMPRSS2 and neighboring MX1 genes. Further, these five minor alleles of SNPs were related with a lower risk of severe COVID-19 infection and higher blood MX1 expression. Importantly, this suggests that MX1 may be a therapeutic target and that host genetic variables can affect the clinical manifestations of COVID-19 [42, 43]. In fact, a substantial association between the COVID-19 case fatality and a nonsynonymous mutation in the TMPRSS2 V197M allele has been demonstrated. In East Asian countries, this was linked to decreased case fatality rates [44].

Additionally, several mutations and polymorphisms have been implicated with respect to COVID-19 co-morbidities. Notably, findings from an Italian study involving the ACE2 eQTLs observed in COVID-19 patients suggest a link between ACE2 genetic variation and neurological disorders [31]. It has also been found that patients with type 2 diabetes may be at a higher risk of SARS-CoV-2 mortality owing to epigenetic changes in the rs13015258-C allele of TMPRSS2 [42]. In African/African Americans, ACE2 polymorphisms such as p. Arg514Gly have been associated with cardiovascular and pulmonary disease [45]. In French Canadians and the United Kingdom, a relation has been established between the T allele of the ACE2 gene SNP rs2074192 and hypertension in obese adult men, particularly smokers [46].

As a final point, some genetic polymorphisms and mutations bear the potential to guide the development and use of related medications. In point of fact, numerous well-studied medications, including acetaminophen (paracetamol) and curcumin, have been demonstrated to inhibit the production of TMPRSS2 in human cells [30]. In India, in the face of the spreading SARS-haplotype-dependent CoV-2, improvement of drug therapy may potentially provide aid for those with the most prevalent haplotypes [47]. Increased attention to polymorphisms in ACE2 or TMPRSS2 may aid in determining the most successful COVID-19 therapy regimen (i.e., hydroxychloroquine and camptothecin) [45].

### Study limitations and prospects

This study had some limitations, owing to the urgency of the COVID-19 epidemic. Firstly, we did not perform a meta-analysis, utilizing forest plots, given the expected heterogeneity of the study design, analytic model (which includes both COVID-19 patients and healthy subjects from the general population), and measurements of ACE2 or TMPRSS2 gene polymorphisms or mutations. Secondly, based on the diversity of included studies, we did not analyze study bias. In future, additional large-sample, multicenter clinical studies are required to validate our findings.

## Conclusion

Genetic diversity may be related to differences between individuals, with regard to infection prognosis, disease severity, and response to various types of pharmacological and other forms of therapy, such as oxygen. Although numerous research efforts have focused on identifying and developing effective pharmacologic strategies, precision medicine holds promise to substantially guide elucidating the role of epigenetic variability in COVID-19. In summary, our findings indicate that ACE2 and TMPRSS2 genes not only play crucial roles in the onset, development, and prognosis of SARS-CoV-2 infections, but are all strongly correlated to susceptibility, intensity, and clinical outcomes of COVID-19. Overall, these findings provide the new insights that will guide future development of personalized drugs and vaccines for COVID-19.

## Data Availability

The authors’ state that all information provided in this article can be shared.
